# Honorary authorship in high-impact journals in anaesthesia and pain medicine

**DOI:** 10.1177/20494637211023526

**Published:** 2021-06-17

**Authors:** Roshni HS Matawlie, Jamie RJ Arjun Sharma, Judith D de Rooij, Geetanjali Sardjoe Mishre, Frank JPM Huygen, Pravesh S Gadjradj

**Affiliations:** 1 Department of Neurosurgery, Leiden University Medical Center, Leiden, The Netherlands; 2 Center for Pain Medicine, Erasmus University Medical Center (Erasmus MC), Rotterdam, The Netherlands

**Keywords:** Honorary authorship, survey, pain, anaesthesia

## Abstract

Enlisting an author on a published paper, whose input was insufficient, is called honorary authorship. The aim of this study is to assess the proportion of honorary authorship in the field of pain medicine. Data were collected from seven high-impact journals. Corresponding authors were sent a survey regarding their awareness on authorship guidelines, the decision-making in authorship and specific contributions made to the surveyed article. We identified two types of honorary authorship: (1) self-perceived honorary authorship, which is measured by asking the corresponding author if honorary authorship was present according to their opinion and (2) International Committee of Medical Journal Editors (ICMJE)-defined honorary authorship, which is honorary authorship based on the guidelines. In total, 1051 mails were sent and 231 responded, leading to a response rate of 22.0%. 81.3% of the respondents were familiar with the ICMJE authorship guidelines, while 59.6% were aware of the issue of honorary authorship. 13.3% of the respondents were employed at a department in which the senior member is automatically included on all manuscripts. The ICMJE-defined honorary authorship was 40%, while self-perceived honorary authorship was 13.5%. There seems to be a high awareness of the ICMJE guidelines among corresponding authors in the field of Pain Medicine. Despite this high awareness, a high proportion of journal articles had honorary authorship, suggesting that authorship guidelines fail to be applied in a significant proportion of the literature.

The International Committee of Medical Journal Editors (ICMJE) developed a guideline which recommends that authorship should be allocated upon fulfilling four criteria:

‘(1) substantial contributions to the conception or design of the work; or the acquisition, analysis, or interpretation of data for the work;
(2) drafting the work or revising it critically for important intellectual content;
(3) final approval of the version to be published;
(4) AND agreement to be accountable for all aspects of the work in ensuring that questions related to the accuracy or integrity of any part of the work are appropriately investigated and resolved’.^1^ Enlisting an author on a published paper, whose input was insufficient, is called honorary authorship (HA). The aim of this pilot study is to get an overview of HA in high-impact journals in pain medicine.

Seven journals with the highest impact factor in the field of Pain Medicine were screened for published articles in the year 2017. Eligible were articles with more than one author and if there was an available e-mail address. Correspondence on manuscripts was excluded. This survey contained 21 questions about the demographics, the awareness on authorship guidelines, decision-making in authorship and specific contributions to the surveyed article.^2–4^ Authors were asked if one or more authors only performed tasks from a list of tasks, for example, proofreading, performing cases, and nothing else related to the manuscript preparation, study design or data analysis. This was defined as ICMJE-defined HA. The survey ended with asking if the respondents felt that according to them, one or more of their coauthors did not deserve HA. This was defined as self-perceived HA. The survey was distributed using SurveyMonkey (Palo Alto, CA). Authors were mailed in 2018 and reminders were sent to increase the response rate.

In total, 1051 mails were sent, with 231 responses leading to a response rate of 22.0%. Table 1 gives an overview of the responses on the survey. 81.3% of the respondents were familiar with the ICMJE authorship guidelines. 59.6% was aware of the issue of HA versus 40.4% being unaware of HA. The vast majority of the responders were first author and corresponding author of the surveyed article (66.5%), of which 73% states that they wrote all or most of the articles. 52.6% decided as a group on the order of the authorship and most of the responders used the amount of contribution of each author to determine the order of authorship (46.7%). There were rarely any suggestions to include an HA (9.3%) and hardly any senior members were automatically included (13.3%), as the latter was mostly seen as unjust (69.8%).

**Table 1.** Overview of answers on the survey.

	n (%)		n (%)
Awareness of the ICMJE authorship guidelines	230	Self-perceived HA	230
Yes	187 (81.3)	Regional Anesthesia and Pain Medicine	1 (5.0)
No	43 (18.7)	The Clinical Journal of Pain	1 (3.6)
		The Journal of Pain	5 (13.9)
Awareness of HA	223	Pain Medicine	8 (19.0)
Yes	133 (59.6)	Pain Practice	4 (46.4)
No	90 (40.4)	Pain	3 (9.1)
		The European Journal of Pain	9 (20.9)
Automatic inclusion of a senior member of your department	226	Funding of the study	231
		(Pharmaceutical) Industry	11 (4.8)
Yes	30(13.3)	University sponsored (e.g. grant)	82 (35.5)
No	187(82.7)	No funds obtained	95 (41.1)
Don’t know	9(4.0)	Other	45 (19.5)
If so, do you feel this is justified?	136	Decided the order of authorship	230
Never justified	55 (40.4)	First author	65 (28.3)
Rarely justified	40 (29.4)	Senior author	36 (15.7)
Sometimes justified	23 (16.9)	Authors decided as a group	121 (52.6)
Most of the time justified	12 (8.8)	The funding source of this study	2 (0.9)
Always justified	6 (4.4)	Other	6 (2.6)
Suggestions for including an HA	226	Did any of your coauthors perform only *one or more* of enlisted non-authorship tasks, and nothing else related to the manuscript preparation, study design or data analysis?	230
Yes	21 (9.3)		
No	205 (90.7)		
Position among the authors	230	Regional Anesthesia and Pain Medicine	6 (30.0)
First author and corresponding author	153 (66.5)	The Clinical Journal of Pain	9 (32.1)
First authors but not corresponding author	2 (0.9)	The Journal of Pain	16 (44.4)
Corresponding author but not first author	21 (9.1)	Pain Medicine	20 (47.6)
Senior author and corresponding author	50 (21.7)	Pain Practice	13 (46.4)
Senior author but not corresponding author	4 (1.7)	Pain	12 (36.4)
		The European Journal of Pain	16 (37.2)
Criteria used to decide the order of authorship	227	If ‘yes’, which performed tasks	231
		Supervising/recruiting coauthors	32 (13.9)
In the order of the amount each contributed	106 (46.7)	Obtaining funding or material support	26 (11.3)
In alphabetical order	1 (0.4)	Recruiting study subjects	41 (17.7)
In the order of the amount each contributed, except the last author, who is the most senior in the group but did not contribute to the study	9 (4.0)	Performing cases used in the study	28 (12.1)
		Contributing illustrations	12 (5.2)
		Reviewing the manuscript	75 (32.5)
In the order of the amount each contributed, except the last author, who provided the concept, supervision and responsibility for all working steps of the project	98 (43.2)	Approving the manuscript before submission	68 (29.4)
		Signing statement of copyright transfer to journal	58 (25.1)
Other	13 (5.7)		

The ICMJE guideline–based HA was 40% versus the self-perceived HA being 13.5%. The most selected non-authorship tasks were reviewing and approving the manuscript before submission (61.9%).

This pilot study is the first study that gives an overview of HA in the pain medicine literature. We showed a guideline-based HA of 40% and a self-perceived HA of 13.5%. This difference in self-perceived and guideline-based HA is also visible in other studies.^2,4,5^ A limitation of this study can be the response rate of 22%, which might give a biased estimation of the proportion of HA.

With the high awareness of the ICMJE guidelines and the understanding of HA, the prevalence of guideline-based HA is high. This suggests that researchers should gain clarification of the usage of the ICMJE criteria’s by creating an open culture to discuss authorships in research groups.
